# Supplementary material for: A geometric graph-based deep learning model for drug-target affinity prediction
Source: BMC Bioinformatics. 2025 Dec 18;27:19. doi: 10.1186/s12859-025-06347-2 (PMC12831358; doi:10.1186/s12859-025-06347-2)
Supplement: Supplementary file 1 — Supplementary Information 1. [file 12859_2025_6347_MOESM1_ESM.pdf]

Supporting Information
A Geometric Graph-Based Deep Learning Model for Drug-Target Affinity Prediction

Md Masud Rana[1*], Farjana Tasnim Mukta[1], and Duc Duy Nguyen[2*]
[1] Department of Mathematics, Kennesaw State University, Kennesaw, GA 30144, USA
[2] Department of Mathematics, University of Tennessee, Knoxville, TN 37996, USA

1 Model hyper-parameters

To identify the optimal kernel size and loss function weight parameter (α), we performed a systematic hyperparameter search using five-fold cross-validation (CV) on the validation set, while keeping all other model parameters fixed as specified in the main text. Specifically, we employed the RepeatedKFold strategy from scikit-learn with five repeats, and repeated the entire CV procedure three times, reporting the average performance across runs. For kernel size optimization, we fixed $\alpha = 0.5$ and varied the kernel size from 3 to 9. As shown in Figure S1, a kernel size of 7 achieved the highest Pearson correlation coefficient. Using this optimal kernel size, we next optimized α by varying its value between 0.1 and 0.9 in increments of 0.1. As illustrated in Figure S2, the best performance was obtained with $\alpha = 0.7$.

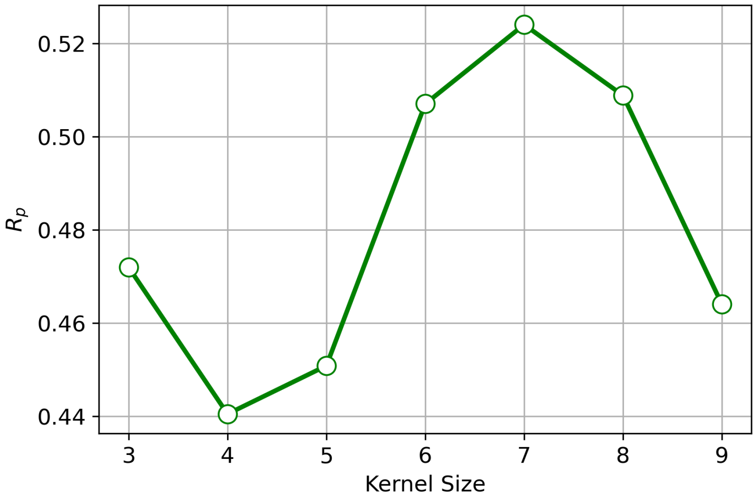

Figure S1: 5-fold cross-validation results of DeepGGL in Pearson correlation (R_p) for varying kernel size. The kernel size of 7 shows the best performance.

2 Results on independent test sets

Scatter plots of the mean predicted values of five independent runs versus experimentally measured binding affinities on the two external datasets, the CSAR NRC-HiQ set and the PDBbind v2019 'hold-out set', are presented in Figure S3.

3 Ablation model architecture and results

Architectures of various ablation models are provided in Figure S4 and their performance on four benchmark test sets is listed in Table S1.

*Corresponding author(s): mrana10@kennesaw.edu; ducnguyen@utk.edu

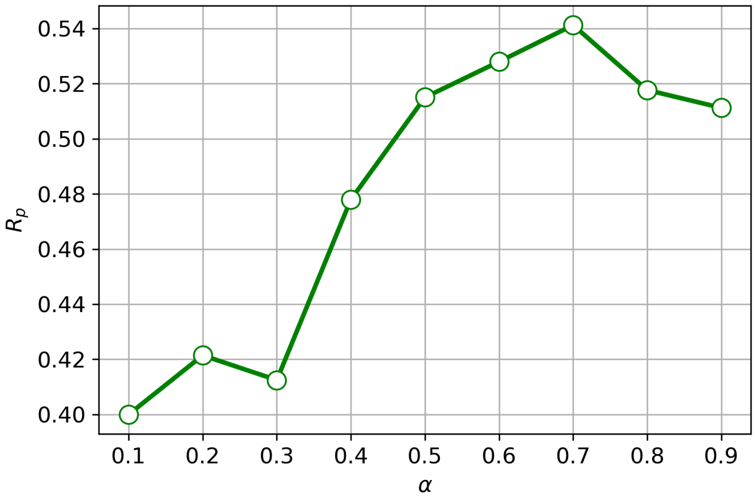

Figure S2: 5-fold cross-validation results of DeepGGL in Pearson correlation (R_p) for varying loss function weight parameter α. The value $\alpha = 0.7$ shows the best performance.

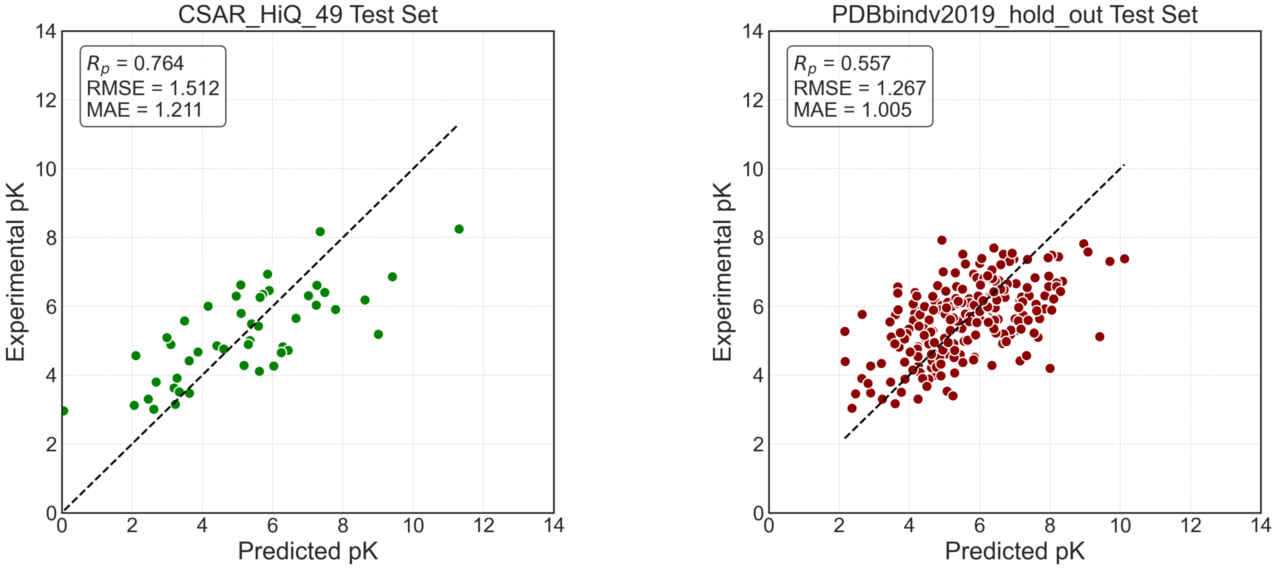

Figure S3: Prediction results of DeepGGL on CSAR-NRC-HiQ (left) and PDBbind v2019 holdout (right) test sets. Each point shows the mean predicted pK for a complex, averaged over five independent training runs, versus its experimentally determined pK.

Table S1: Model ablation study: Pearson correlation coefficient (R_p) across four benchmark datasets.

Model Variant	CASF 2016	CASF 2013	CSAR-HiQ	v2019-holdout
DeepGGL	**0.868**	0.844	0.764	**0.557**
Without residual block	0.857	0.835	0.767	0.547
Without attention mechanism	0.863	**0.845**	**0.769**	0.544
Without attention and residual block	0.861	0.840	0.756	0.507

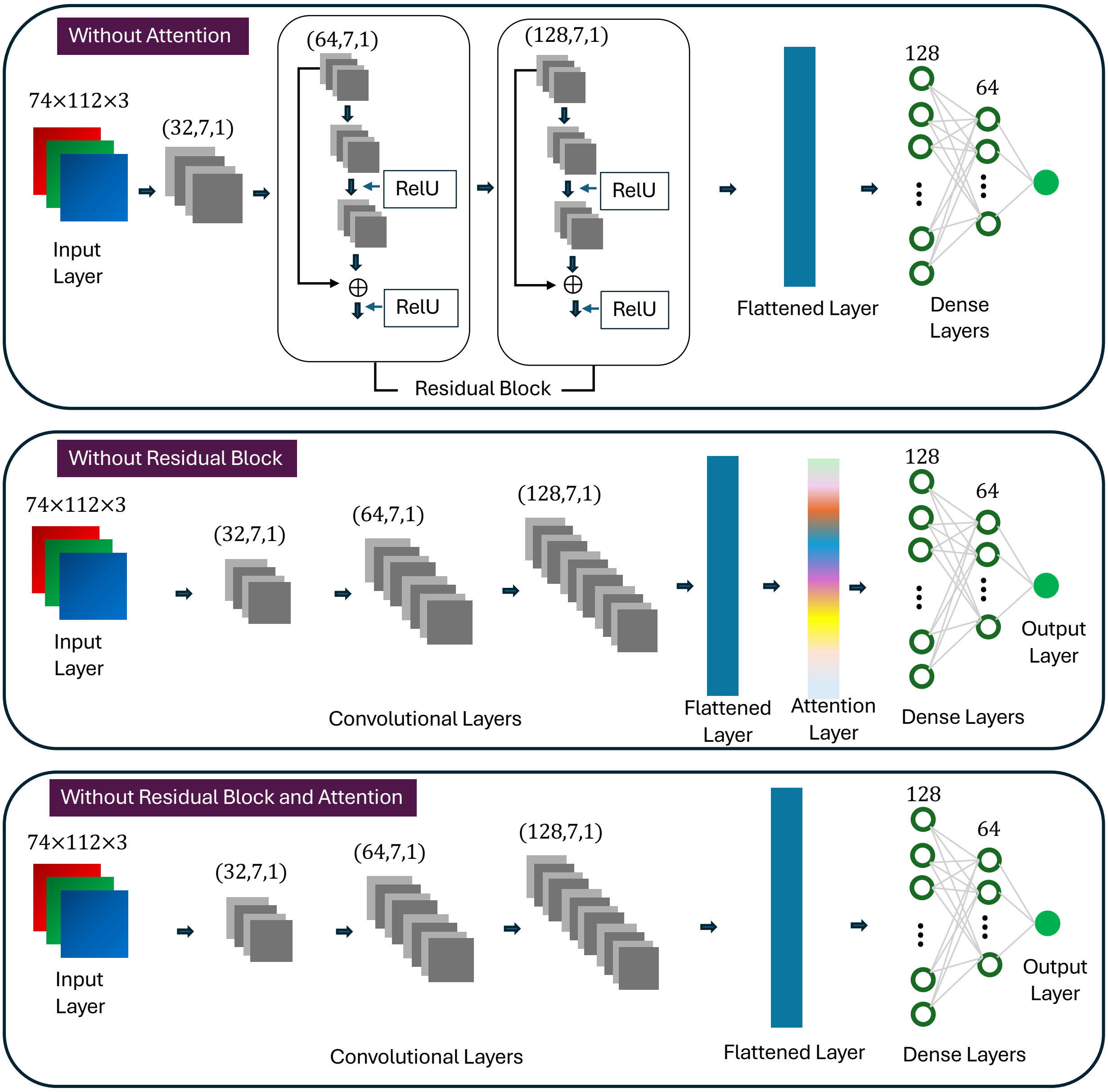

Figure S4: DeepGGL ablation model architectures.

4 The state-of-the-art methods for comparison

References

[1] Zhenyu Meng and Kelin Xia. Persistent spectral–based machine learning (perspect ml) for protein-ligand binding affinity prediction. *Sci. Adv.*, 7(19):eabc5329, 2021.

[2] Duc Duy Nguyen and Guo-Wei Wei. Agl-score: algebraic graph learning score for protein–ligand binding scoring, ranking, docking, and screening. *J. Chem. Inf. Model.*, 59(7):3291–3304, 2019.

[3] Maciej Wójcikowski, MichałKukiełka, Marta M Stepniewska-Dziubinska, and Pawel Siedlecki. Development of a protein–ligand extended connectivity (plec) fingerprint and its application for binding affinity predictions. *Bioinformatics*, 35(8):1334–1341, 2019.

[4] Zechen Wang, Liangzhen Zheng, Yang Liu, Yuanyuan Qu, Yong-Qiang Li, Mingwen Zhao, Yuguang Mu, and Weifeng Li. Onionnet-2: a convolutional neural network model for predicting protein-ligand binding affinity based on residue-atom contacting shells. *Frontiers in chemistry*, 9:753002, 2021.

Table S2: State-of-the-art machine learning-based scoring functions used in the study for comparison.

Method	Year	Feature	Training Set	ML Algorithm
PerSpect [1]	2021	algebraic topology and persistent spectral graph	v2016 refined set	GBT
AGL-Score [2]	2019	algebraic graph	v2016 refined set	GBT
PLEC-Linear [3]	2019	extended connectivity fingerprint	v2016 general set	Linear Regression
OnionNet-2 [4]	2021	contact numbers between protein residues and ligand atoms in multiple distance shells	v2019 general set	CNN + DNN
EIGN [5]	2025	interaction graph structre of complex	v2016 general set	GNN
EGNA [6]	2023	protein, ligand, and their interaction graphs, constructed from different regions	v2016 general set	GNN
DeepTGIN [7]	2024	sequence and graph isomorphism	v2020 general set	Transformer + GIN
OnionNet [8]	2019	hierarchical distances	v2016 general set	CNN + DNN
Pafnucy [9]	2018	3D voxel representation	v2016 general set	CNN
CAPLA[10]	2023	sequence-level information of both protein and ligand	v2016 general set	Cross-attention, FNN
DeepDTAF [11]	2021	sequence and structural information (SSEs and physicochemical) of global protein and protein pocket and ligand SMILES	v2016 general set	CNN + FNN

[5] Dinghai Yang, Linai Kuang, and An Hu. Edge-enhanced interaction graph network for protein-ligand binding affinity prediction. *PloS one*, 20(4):e0320465, 2025.

[6] Chunqiu Xia, Shi-Hao Feng, Ying Xia, Xiaoyong Pan, and Hong-Bin Shen. Leveraging scaffold information to predict protein–ligand binding affinity with an empirical graph neural network. *Brief. Bioinform.*, 24(1), 2023.

[7] Guishen Wang, Hangchen Zhang, Mengting Shao, Yuncong Feng, Chen Cao, and Xiaowen Hu. Deeptgin: a novel hybrid multimodal approach using transformers and graph isomorphism networks for protein-ligand binding affinity prediction. *Journal of Cheminformatics*, 16(1):147, 2024.

[8] Liangzhen Zheng, Jingrong Fan, and Yuguang Mu. Onionnet: a multiple-layer intermolecular-contact-based convolutional neural network for protein–ligand binding affinity prediction. *ACS omega*, 4(14):15956–15965, 2019.

[9] Marta M Stepniewska-Dziubinska, Piotr Zielenkiewicz, and Pawel Siedlecki. Development and evaluation of a deep learning model for protein-ligand binding affinity prediction. *Bioinformatics*, 34(21):3666–3674, 2018.

[10] Zhi Jin, Tingfang Wu, Taoning Chen, Deng Pan, Xuejiao Wang, Jingxin Xie, Lijun Quan, and Qiang Lyu. Capla: improved prediction of protein–ligand binding affinity by a deep learning approach based on a cross-attention mechanism. *Bioinformatics*, 39(2):btad049, 2023.

[11] Kaili Wang, Renyi Zhou, Yaohang Li, and Min Li. Deepdtaf: a deep learning method to predict protein–ligand binding affinity. *Briefings in Bioinformatics*, 22(5):bbab072, 2021.
